# Neural Stem Cells Overexpressing Nerve Growth Factor Improve Functional Recovery in Rats Following Spinal Cord Injury via Modulating Microenvironment and Enhancing Endogenous Neurogenesis

**DOI:** 10.3389/fncel.2021.773375

**Published:** 2021-12-02

**Authors:** Lei Wang, Sujie Gu, Jinlu Gan, Yi Tian, Fangcheng Zhang, Hongyang Zhao, Deqiang Lei

**Affiliations:** Department of Neurosurgery, Union Hospital, Tongji Medical College, Huazhong University of Science and Technology, Wuhan, China

**Keywords:** recovery, cell transplantataion, spinal cord injury, nerve growth factor, neural stem cells (NSCs), microenviroment, endogenous neurogenesis

## Abstract

Spinal cord injury (SCI) is a devastating event characterized by severe motor, sensory, and autonomic dysfunction. Currently, there is no effective treatment. Previous studies showed neural growth factor (NGF) administration was a potential treatment for SCI. However, its targeted delivery is still challenging. In this study, neural stem cells (NSCs) were genetically modified to overexpress NGF, and we evaluated its therapeutic value following SCI. Four weeks after transplantation, we observed that NGF-NSCs significantly enhanced the motor function of hindlimbs after SCI and alleviated histopathological damage at the lesion epicenter. Notably, the survival NGF-NSCs at lesion core maintained high levels of NGF. Further immunochemical assays demonstrated the graft of NGF-NSCs modulated the microenvironment around lesion core via reduction of oligodendrocyte loss, attenuation of astrocytosis and demyelination, preservation of neurons, and increasing expression of multiple growth factors. More importantly, NGF-NSCs seemed to crosstalk with and activate resident NSCs, and high levels of NGF activated TrkA, upregulated cAMP-response element binding protein (CREB) and microRNA-132 around the lesion center. Taken together, the transplantation of NGF-NSCs in the subacute stage of traumatic SCI can facilitate functional recovery by modulating the microenvironment and enhancing endogenous neurogenesis in rats. And its neuroprotective effect may be mediated by activating TrkA, up-regulation of CREB, and microRNA-132.

## Introduction

Spinal cord injury (SCI) is a devastating trauma to the central nervous system (CNS), characterized by severe motor, sensory and autonomic dysfunction. In China, there were 10,074 cases of traumatic SCI in 2018 with a total estimated incidence of 66.5 cases per million in population, and the number is still rising (Hao et al., 2021). However, its treatment is still a major problem in this field, and there is no effective cure currently (Fan et al., 2018).

Following SCI, the immediate physical impact and secondary injury (e.g., robust neuroinflammation, neuronal death, axonal collapse, demyelination, and glial scar formation, etc.) result in an inhibitory environment, which hampers neurite regeneration, neural plasticity, and neurogenesis (Hayta and Elden, 2018). Thus, the key of SCI therapy is to modulate the microenvironment and restore the function of damaged axons and neurons (Anderson et al., 2018; Thakor et al., 2018).

The recent investigations suggest that neurotrophins can overcome the failure of axon regeneration in the complete SCI (Keefe et al., 2017; Anderson et al., 2018). Nerve growth factor (NGF) is a classic neurotrophin in the spinal cord, and it is known to exert a neuroprotective effect on the injured spinal cord (Sofroniew et al., 2001). Song et al. (2017) administrated NGF via ultrasound-mediated nanobubble destruction in acute spinal cord injury. They observed significant restoration of motor function, attenuated histological damage, less neuronal loss, and apoptosis after treatment in SCI rats. However, most NGF in the nanobubbles would be released in 48 h, and repeat injections were required to maintain high levels of NGF at the lesion site even the advanced nanotechnology was adopted (Song et al., 2017). Thus, the sustained delivery of NGF to CNS either across the blood-brain barrier or via intrathecal infusion is still challenging, which is a bottleneck for SCI treatment.

Herein, we overexpressed NGF in spinal cord-derived neural stem cells (NSCs) and transplanted NGF-NSCs in the lesion core of the contusive SCI rat. The NGF-NSCs was acted as cell deliverers to release NGF in the injured site consistently. This study aims to determine whether the transplanted NGF-NSCs can facilitate functional recovery after SCI and explore its underlying mechanism.

## Materials and Methods

### Animals

Female Sprague-Dawley (SD) rats (220–250 g) and 14-day pregnant SD rats were obtained from the experimental animal center of Tongji medical college. The rats were housed in cages at 22°C and 55% humidity under a 12:12 h light-dark cycle with free access to food and water. All procedures involved in experimental animals were approved by the Committee of Experimental Animals of Union Hospital per the guidelines of China Council on Animal Care and Use.

### Cell Culture and Transfection

Neural stem cells were obtained from the spinal cord tissues of day 14 embryonic SD rats, which were isolated and cultured per established protocol (Lei et al., 2009). Briefly, spinal cord tissues were separated and cut into small pieces, and then the pieces were digested with trypsin to collect cell suspension. After centrifuging at 1,500 rpm for 6 min, the supernatant was discarded. The acquired NSCs were plated on cell culture flasks with a complete medium (Procell, Wuhan, China) containing Dulbecco’s Modified Eagle Medium/F-12, 20 ng/mL epidermal growth factor, 20ng/mL basic fibroblast growth factor, 2% B-27 supplement, 1% Penicillin-Streptomycin and 4mmol/L L-glutamine. The NSCs were cultured in a chamber with 5% CO_2_ at 37°C and passaged 1–2 times 1 week. The lentiviral vectors overexpressing NGF (pLVX-IRES-ZsGreen-rat NGF) were synthesized by Biobuffer Biotech Service (Wuhan, China). They were used to transfect the NSCs at 3rd passage and exponential growth period.

For the differentiation *in vitro*, NGF-NSCs were seeded in the mediums composed of DMEM/F12 (Procell), 1% N2 supplement (Thermo Fisher Scientific, Waltham, WI, United States), 2mmol/L L-glutamine (Thermo Fisher Scientific), 1% Penicillin-Streptomycin (Aladdin, Shanghai, China) and 1% Fetal Bovine Serum (Excell Bio, Shanghai, China) for 7 days.

### Immunocytochemistry and Immunohistochemistry of Cells

Immunocytochemistry of cell-specific markers (including Nestin, CNPase, GFAP, and β-tubulin3) was performed in NGF-NSCs neurospheres to identify NSCs after transfection. Briefly, the neurospheres were harvested and seeded on the coverslips pre-coated with poly-l-lysine and laminin in the 24-wells plates overnight. And then, the cells were fixed with 4% paraformaldehyde (PFA, Thermo Fisher Scientific) and permeabilized with 0.1% Triton (BD Biosciences). Following the blocking by normal goat serum, primary antibodies of Nestin (1:100, Proteintech, Wuhan, China), CNPase (1:200, Proteintech), GFAP (1:200, Cell Signaling Technology, Shanghai, China), and β-tubulin3 (1:100, Cell Signaling Technology) were added to the samples and incubated overnight at 4°C. After washing with PBS the next day, the cell samples were incubated with appropriate fluorescent secondary antibody (1:100, Boster), and then DAPI (Beyotime) was used to label the nucleus.

To verify the expression of NGF and investigate the differentiation of NGF-NSCs cells, we performed immunohistochemistry in the neurospheres and differentiated NSCs. Similar to the immunocytochemistry, the samples were first fixed with 4% PFA (Thermo Fisher Scientific) and permeabilized with 0.1% Triton (BD Biosciences). Subsequently, H_2_O_2_ solution and normal goat serum were applied to incubate the cells to block the samples. Primary antibodies to Nestin (1:300, Proteintech), CNPase (1:200, Proteintech), GFAP (1:400, Cell Signaling Technology), and β-tubulin3 (1:200, Cell Signaling Technology) were added to the samples, which were then incubated with the appropriate horseradish peroxidase-conjugated secondary antibody. Finally, the cells were stained with freshly prepared diaminobenzidine tetrahydrochloride (DAB, Proteintech) and counterstained with hematoxylin (Sigma-Aldrich).

### Spinal Cord Injury Model and Neural Stem Cells Transplantation

After adaptive feeding for 7 days, a total of 45 rats were randomly divided into two groups, the control group (*n* = 5) and the SCI group (*n* = 40). Then the SCI rats were intraperitoneally injected with 1.5% pentobarbital solution (65 mg/kg) for anesthesia before surgery. The back skin and subcutaneous tissues of the surgery zone were cut longitudinally on the operating table. At the level of T10, the spinous process and lamina were removed to expose the spinal cord. According to Allen’s simple strike model, the SCI strike device (Calvin, Nanjing, China) was used to hit the cord (impact rod weight: 20g, drop height: 2.5 cm). If the rat had spinal cord congestion, spasmodic shaking of both lower limbs, and tail spasmodic swinging after striking, the model was considered successful. The muscles and skins of rats were sutured by layers. Intraperitoneal injection of penicillin was given for 3 days after surgery, and manual bladder evacuation was performed twice every day until the restoration of the bladder function. Seven days after SCI, the rats were then randomly divided into sham (*n* = 20) and NGF-NSCs transplantation groups (*n* = 20). In the transplantation group, NGF-modified NSCs were injected by using a microsyringe (Hamilton, Bonaduz, Switzerland). In brief, 5 ul cells suspension containing 5^∗^10^4^of NGF modified NSCs were injected at the lesion epicenter after anesthesia. In the sham group, the 5 ul phosphate buffered saline (PBS, Sigma-Aldrich, St. Louis, MO, United States) was injected in the same site. Five rats per group were randomly selected and sacrificed for further protein and RNA analysis every week after transplantation. The locomotor function of hind limbs was evaluated weekly until sacrifice.

### Behavioral Assessments

Basso-Beattie-Bresnahan (BBB) scale, inclined plate test, and grid walking test were used to evaluate the functional recovery of hind limbs’ locomotion. Evaluations were conducted 1 and 7 days after SCI and 7, 14, 21, and 28 days after NSCs transplantation by two well-trained observers blinded to grouping. According to the BBB scale, rats were allowed to move freely in an open field for 4 min, and the average locomotor scores were recorded. As for the inclined plate test, a 6 mm thick rubber pad was placed on the surface of an inclined plane, and rats were placed in a body axis direction perpendicular to the longitudinal axis of the inclined plate. The inclination angle was gradually increased, and the rats were required to stay on the inclined plate for at least 5 s to record the maximum angle. Each rat was measured three times, and the average angle was recorded. In the grid walking tests, the rats were placed on an elevated metal grid with each grid cell 2.5 × 2.5 cm^2^. The grid was 30 × 80 cm^2^ and enclosed by transparent plexiglass walls with two openings. The performance of experimental rats was scored by counting the number of placing hindlimbs not on the wireframe to hold their body weight while moving along the grid every 20 steps (10 steps per hind limb). If the rat’s hind limbs couldn’t support the body mass, the number of incorrect steps is 20.

### RNA Extraction and Reverse Transcription-Quantitative Polymerase Chain Reaction

Total RNA of tissues was isolated with TRIzol reagent (Aidlab Biotechnologies, Beijing, China). After lysing for 5 min, chloroform (Thermo Fisher Scientific) was added to the mixed solution. Then the supernatant was mixed with isopropyl alcohol and centrifuged for 10 min at 12,000 rpm and 4°C. After washing with 75% alcohol twice, the precipitated RNA was dissolved by DEPC-water and stored at −4°C. Reverse-transcription was performed, and the synthesized cDNAs were amplified and quantified using the QuantStudio 6 PCR system (Thermo Fisher Scientific). Glyceraldehyde 3-phosphate dehydrogenase (GAPDH) was used as an internal control. All primer sequences are shown in Supplementary Table 1. The calculation and quantification of gene expression were based on the 2^–^^ΔΔ^ ^Ct^ method.

### Protein Extraction and Western Blotting Analysis

The proteins of primarily cultured NSCs or spinal cord tissues from SCI rats were isolated by RIPA lysis buffer (Beyotime, Shanghai, China), and the concentrations were determined by a BCA detection kit (Beyotime). About 20ug proteins were loaded and electrophoresed onto 12% SDS polyacrylamide gel (GE Healthcare Bioscience, Marlborough, MA, United States) and then transferred to polyvinylidene fluoride (PVDF) membrane (MilliporeSigma, Burlington, MA, United States). The membranes were blocked by 5% non-fat milk for 60 mins at 37°C and were then incubated with anti-NGF monoclonal antibody (1:1000, Abcam, Cambridge, MA, United States), anti-CREB (1:1000, Abcam), anti-GDNF (1:500, Abclonal, Wuhan, China), anti-BDNF (1:1000, Abcam), anti-VEGF (1:1000, Boster, Pleasanton, CA, United States) and anti-β-actin (1:5000, Boster) overnight at 4°C. The primary antibody incubation was followed by incubation with a secondary peroxidase-conjugated anti-rabbit (1:5000, Boster) or anti-mouse antibody (1:5000, Boster) at room temperature for 2 h. The detection of signal was conducted using Bryo ECL kit (Bytotime), and the measurement of proteins’ expression was done by Image J (Joonas “Regalis” Rikkonen, Version 1.8.0).

### Immunofluorescence Staining of Spinal Cord

The injured spinal cords were harvested, and the sections were fixed with 4% paraformaldehyde (PFA) (Thermo Fisher Scientific) in PBS at room temperature for 3 h, followed by cryoprotection with 6% sucrose/PBS overnight and embedding in optimal cutting temperature compound to obtain 5 μm thick sections. Representative cross-sections of spinal cord were randomly picked up from SCI epicenter and peri-lesion segments (3 mm caudal to the epicenter), which contained similar morphology of the target regions without major tissue artifact. Standardized immunohistochemical (IHC) reactions were performed in a single-blinded method per established protocol. Briefly, the sections were blocked with 10% goat serum (Boster) diluted in PBS for 1 h, followed by incubation with primary antibodies overnight at 4°C. The following primary antibodies were used: anti-Nestin (1:100, Proteintech, Wuhan, China), anti-CNPase (1:200, Proteintech), anti-GFAP (1:200, Cell Signaling Technology, Shanghai, China), anti-β-tubulin3 (1:100, Cell Signaling Technology), anti-NGF (1:250, Abcam), anti-TrkA and p-TrKA (1:300, immunoway). Prior to being incubated with fluorescent secondary antibody (1:100, Boster), the sections were rinsed with PBST (Sigma-Aldrich) three times (3 min each time). Then DAPI (Beyotime) was used to label the nucleus on the specimen. In each visual field, targeted marker-positive areas were calculated under a fluorescence microscope at 200X magnification, and the Image J software was applied to count the positive areas.

### Electron Microscopy of the Myelin Sheath

After the rats were anesthetized, 1-mm^3^ fresh tissue from the injury epicenter was harvested and fixed with 2.5% glutaraldehyde (Thermo Fisher Scientific) for 24 h at 4°C. Then samples were postfixed with 1% Osmium tetroxide (Tedpella, Redding, CA, United States) in 0.1M Phosphate buffer (pH 7.4) and dehydrated gradually in ethanol at room temperature. After embedding with EMBed 812 (Ted Pella), samples were kept at 37°C overnight and moved to 65°C to polymerize for at least 48 h before cutting into 60 to 80 nm sections with the ultramicrotome (Leica, Germany). Uranium acetate (SPI- Chem, Westchester, PA, United States) and lead citrate (SPI-Chem) were used to stain in sequence. Finally, samples were observed, and images were captured under a transmission electron microscope (Hitachi, Japan).

### Statistical Analysis

All data in this study are presented as the mean ± standard derivation (SD) of at least three replicates for each experiment. The statistical analyses were conducted in Graphpad Prism (FreeImage, Version 8.4.2). One-way and two-way analysis of variance (ANOVA) test with *post hoc* were used to evaluate differences of measurement data in groups. The data of behavioral assessments, including BBB scores, inclined plane test, and grid waking test, were processed with two-way ANOVA with *post hoc*. *p* value < 0.05 was considered statistically significant.

## Results

### Establishment of NGF-Modified NSCs and Quality Evaluation

**FIGURE 1:**
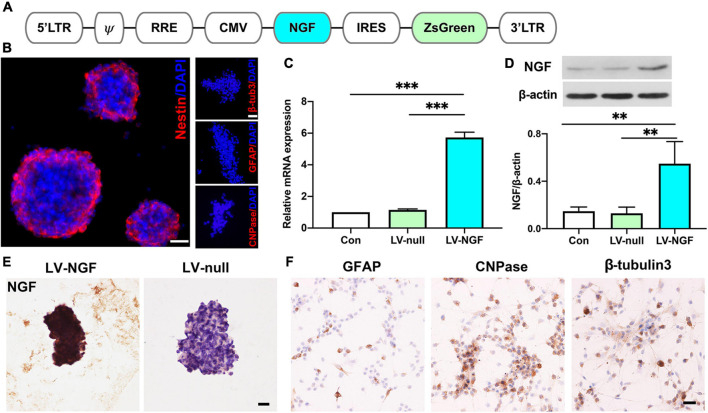
Establishment of NGF-modified NSCs and quality evaluation. **(A)** The design of lentiviral vectors expressing NGF (pLVX-IRES-ZsGreen-rat NGF). **(B)** NGF-modified NSCs highly expressed nestin and they had no immunoactivity with GFAP, CNPase, and β-tubulin3. **(C)** RT-PCR showed the mRNA level of NGF was significantly enhanced in NGF-NSCs. **(D)** Western blotting further confirmed the elevated protein concentration of NGF in NGF-NSCs. **(E)** Representative immunocytochemical images demonstrated high levels of NGF in NGF-NSCs neurospheres. **(F)** NGF-NSCs could differentiate into β-tubulin3-positive neurons, GFAP-positive astrocytes, and CNPase-positive oligodendrocytes. Scale bar = 20 μm. **p < 0.01 and ***p < 0.001.

Lentiviral vectors expressing NGF (pLVX-IRES-ZsGreen-rat NGF) were designed as Figure 1A and transfected into neural stem cells obtained from day 14 rat embryonic spinal cord. As the most important marker of stem cells, nestin was positive in NGF- NSCs, and they had no immunoactivity with GFAP, CNPase, and β-tubulin3 (Figure 1B). In comparison with the NSCs carrying blank lentiviral vectors and naïve NSCs, RT-PCR and western blot suggested the mRNA and protein levels of NGF in NGF-NSCs increased significantly (*p* < 0.01, Figures 1C,D). The representative immunocytochemical staining of NGF-NSCs neurospheres demonstrated high expression of NGF (Figure 1E). The differentiation assay *in vitro* indicated that NGF-NSCs could maintain the stemness and differentiate into β-tubulin3-positive neurons, GFAP-positive astrocytes, and CNPase-positive oligodendrocytes (Figure 1F).

### Transplantation of NGF-Modified NSCs Improves Functional Recovery of Hindlimbs and Alleviates Histopathological Damage After SCI

**FIGURE 2:**
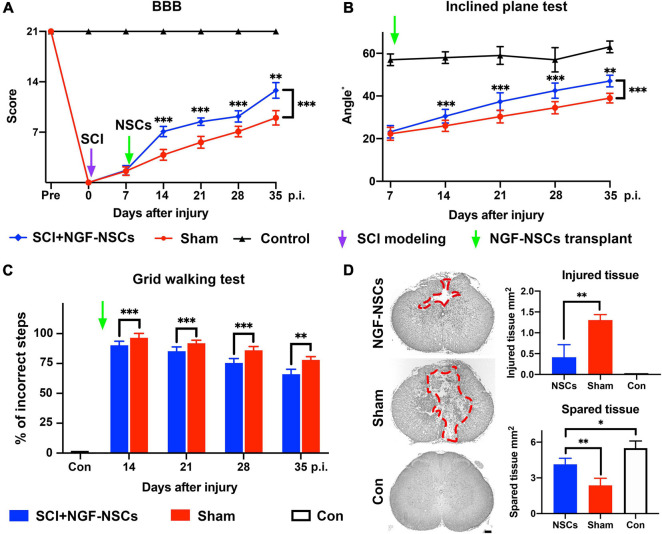
Transplantation of NGF-modified NSCs improved functional recovery of hindlimbs and alleviated histopathological damage after SCI. **(A–C)** After NGF-NSCs transplantation, NGF-NSCs rats exhibited steady and significantly better improvement of hindlimbs’ motor function along time in BBB score **(A)**, inclined plane test **(B)**, and grid walking test **(C)**. **(D)** NGF-NSCs transplantation ameliorated the histopathological damage in the lesion epicenter 4 weeks after treatments. The area of injured tissue was circled by red dash lines in the representative transverse sections. The quantified analysis revealed that NGF-NSCs rats had a smaller area of damaged tissue and a larger size of spared tissue in the lesion site. ^∗^*p* < 0.05, ^∗∗^*p* < 0.01, and ^∗∗∗^*p* < 0.001.

To investigate the protective effect of NGF- NSCs, we established the contusive SCI rat models. We monitored the hindlimbs’ motor function changes via a behavioral battery, including BBB score, inclined plane test, and grid walking test. Notably, we observed significant improvement of functional recovery in the SCI rats receiving NGF- NSCs transplantation over time (two-way ANOVA with *post hoc*, *p* < 0.01 for three tests, Figures 2A–C). Specifically, in the BBB scale, no rats exhibit deficits in locomotion before the injury, and the score decreased dramatically after SCI, which suggested the successful establishment of the SCI model. After cell transplantation, in comparison with the sham group, NGF-NSCs groups demonstrated significant higher BBB scores at post-injury day 14 (*n* = 20/group, *p* < 0.01), day 21 (*n* = 15/group, *p* < 0.01), day 28 (*n* = 10/group, *p* < 0.01) and day 35 (*n* = 5/group, *p* < 0.01, Figure 2A). Meanwhile, the inclined plane tests, another well-known measurement of hindlimb coordinated function, also showed that NGF-NSCs rats were able to maintain a stable body position on the tilted surface with a larger angle along in the subacute and early chronic stages after SCI (two-way ANOVA with *post hoc*, *p* < 0.01, Figure 2B). In the grid walking task, no rats could support their body weight seven days after SCI and the percentage of their incorrect steps at the time was 100% in both groups. One week after cell transplantation, NGF-NSCs rats started to exhibit more rapid progress in the proper placement of hindlimbs when climbing across the grid, and this leading lasted until the end of our observation (two-way ANOVA with *post hoc*, *p* < 0.01, Figure 2C).

To investigate the tissue preservation effect of NGF-NSCs grafts, the representative transverse sections at the lesion epicenter from both groups were qualitatively and quantitatively analyzed for the morphological integrity of neural tissue. The injured tissue was defined as lesioned cord containing injury cavities and severely distorted neural tissue (Figure 2D). As demonstrated in the representative slices, the injured cavities and neural tissue of NGF-NSCs rats were mainly located in the dorsal funiculi and part of the dorsal horn in the gray matter, and the sham rats exhibited worse pathology with larger cavity and profound tissue distortion in the ventral cord (Figure 2D). More importantly, the quantitative analysis of the injured and spared tissues in the lesion epicenter indicated that the damaged tissue area in the NGF-NSCs group was significantly smaller than that in the sham group, and the spared tissue area exhibited the opposite change (one-way ANOVA, *p* < 0.05, Figure 2D).

### Survival and Differentiation of NGF-Modified NSCs After Transplantation and Continuously Expressed High Levels of NGF at the Lesion Core

**FIGURE 3:**
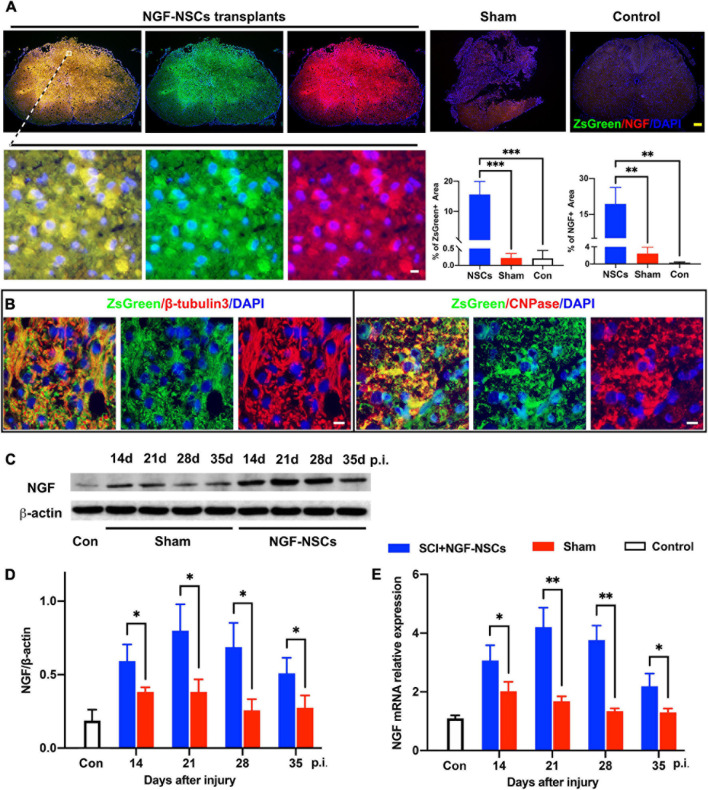
Survival and differentiation of NGF-modified NSCs after transplantation and high levels of NGF at the lesion core. **(A)** Four weeks after NGF-NSCs transplantation, we observed that NGF + /ZsGreen + cells aggregated in the epicenter of the injured spinal cord, and several of them migrated into the peri-lesion area. In comparison, the sham and control rats did not show a positive signal of ZsGreen, and their immunoactivity of NGF was much weaker. **(B)** The transplanted NGF-NSCs marked by ZsGreen expressed β-tubulins3 and CNPase, which indicated the NGF-NSCs could differentiate into neurons and oligodendrocytes. **(C,D)** The western blotting analysis demonstrated that NGF’s protein level was much higher in the NGF-NSCs groups at day 14, 21, 28, and 35 after injury. **(E)** Similarly, the mRNA level of NGF exhibited the same elevation in the three groups. Scale bar, white bar 5 μm; yellow bar 200 μm. ^∗^*p* < 0.05, ^∗∗^*p* < 0.01, and ^∗∗∗^*p* < 0.001.

As showed in Figure 3A, after transplantation, we observed that clusters of NGF-NSCs cells marked by ZsGreen (green signals) aggregated in the epicenter of the injured spinal cord, and several of them were extended into the peri-lesion area. Additionally, NGF (red signal) was co-localized with ZsGreen (green signal) in these cells and they exhibited higher immunoactivity in the sample of the NGF-NSCs group (one-way ANOVA, *p* < 0.05, Figure 3A). In comparison, the positive signal of NGF was much weaker in the control and sham groups (one-way ANOVA, *p* < 0.05, Figure 3A). Notably, we observed that the transplanted NGF-NSCs marked by ZsGreen expressed β-tubulins3 and CNPase (the representative images were shown in Figure 3B), which suggested the NGF-NSCs could differentiate into neurons and oligodendrocytes. Further western blotting and RT-PCR exhibited that NGF’s protein and mRNA levels in both sham and NGF-NSCs groups were upregulated at day 7, 14, 21, and 28 after the graft (student t test, *p* < 0.05, Figures 3C–E). However, compared to the spontaneous NGF expression after SCI in sham rats, the elevation in NGF-NSCs rats was much more significant, and this effect still existed 4 weeks after transplantation (Figures 3C–E). Taken together, our findings suggested the NGF-NSCs could survive, differentiate, and continuously secret NGF at the lesion site after cell transplantation in the subacute and chronic stage of SCI.

### Reduction of Oligodendrocyte Loss and Attenuation of Astrocytosis and Demyelination in the Peri-Lesion Segments After NGF-Modified NSCs Transplantation

**FIGURE 4:**
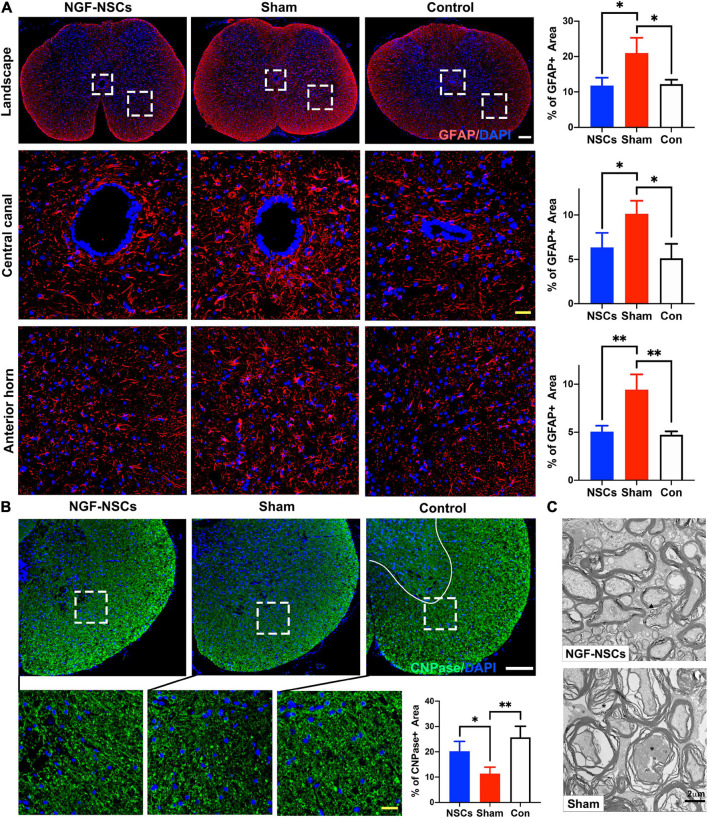
NGF-modified NSCs transplantation reduced the oligodendrocyte loss, attenuated astrocytosis, and demyelination in the peri-lesion segments post SCI. **(A)** Five weeks after SCI, the sham rats exhibited significantly higher expression of GFAP in the landscape, anterior horn, and around the central canal in the peri-lesion segments (3 mm caudal to the lesion epicenter). The graft of NGF-NSCs dramatically attenuated the abnormal GFAP expression in the same regions. **(B)** NGF-NSCs treatment reversed the down-regulation of CNPase in the peri-lesion segments (3 mm caudal to the lesion epicenter). after SCI. **(C)** The representative electoral microscopy of the white matter in the lesion epicenter showed that the integrity of the nerve sheath and corresponding axons were preserved after NGF-NSCs transplantation. Scale bar: white 200 μm; yellow 20 μm. *p < 0.05 and **p < 0.01.

After SCI, the imbalance of microenvironment and progressive secondary injury cascade in the peri-lesion segments directly contribute to sustained and irreversible functional deficits (Oyinbo, 2011; Fan et al., 2018). Thus, we zoomed into the peri-lesion site to check the effect of NGF-NSCs transplantation. Five weeks after SCI, the sham rats exhibited significantly higher expression of GFAP (a cellular marker for the astrocytes) in the anterior horn and around the central canal 3 mm apart from lesion epicenter (one-way ANOVA, *p* < 0.05, Figure 4A). The graft of NGF-NSCs dramatically attenuated the abnormal GFAP expression in the peri-lesion segments, and the corresponding immunoactivity of GFAP in the anterior horn and around the central canal was similar to that in the control rat without SCI (one-way ANOVA, *p* < 0.05, Figure 4A). At the same time, NGF-NSCs treatment reversed the down-regulation of CNPase (a cellular marker for the oligodendrocytes) after injury at the same level, which suggested NGF-NSCs could reduce the loss of oligodendrocytes. As shown in Figure 4B, the healthy spinal cord demonstrated high expression of CNPase in the anterior horn and ventral funiculi, and its expression was significantly reduced in the early chronic stage of SCI (one-way ANOVA, *p* < 0.05). Four weeks after cell transplantation, we observed the restoration of the CNPase expression at the same level in the NGF-NSCs group (one-way ANOVA, *p* < 0.05, Figure 4B). Further electoral microscopy demonstrated that the integrity of the nerve sheath and corresponding axons could be better preserved in the white matter at the lesion epicenter of SCI rats receiving NGF-NSCs (the representative images were shown in Figure 4C).

### Preservation of Motor Neurons and Enhancement of Endogenous Neurogenesis in the Peri-Lesion Segments After NGF-Modified NSCs Transplantation

**FIGURE 5:**
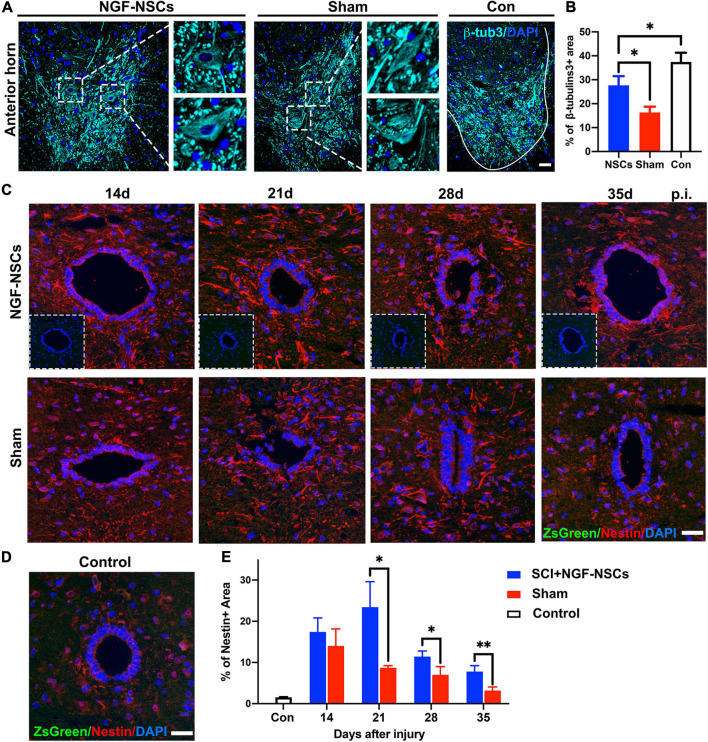
NGF-modified NSCs transplantation protected motor neurons and enhanced endogenous neurogenesis in the peri-lesion segments. **(A,B)** Four weeks after NGF-NSCs transplantation, compared to the sham group, the percentage of β-tubulin3 positive area in the anterior horn was significantly higher, and the neurons maintained physiological morphology. **(C)** Around the central canal in the peri-lesion segments (3 mm caudal to the lesion epicenter), there were no positive signals of ZsGreen and the immunoactivity of nestin was higher in the rat receiving NGF-NSCs along the time. **(D)** In the control rats, the expression of nestin was weak around central canal and there were no ZsGreen + cells. **(E)** The semi-quantification of the percentage of nestin + area demonstrated that high expression of nestin in the peri-lesion segments of NGF-NSCs rats. Scale bar = 20 μm. *p < 0.05 and **p < 0.01.

As reported previously, the re-establishment of local neural circuits is pivotal to the functional recovery after SCI (Han Q. et al., 2019). Thus, we used β-tubulin3, which is constitutively expressed in mature neurons and participated in the timely axon regeneration, to explore the neuroprotective effects of the NGF-NSCs. As shown in Figure 5A, compared to the sham group, the positive area of β-tubulin3 was significantly higher in the anterior horn of the NGF-NSCs group (3 mm caudal to the lesion epicenter) 4 weeks after the transplantation (one-way ANOVA, *p* < 0.05, Figures 5A,B). Additionally, the motor neurons degenerated morphologically after SCI, and the transplantation of NGF-NSCs helped to maintain the neuronal integrity (Figure 5A).

Next, we investigated endogenous neurogenesis in the peri-lesion segments. The analysis was performed on spinal sections 3 mm caudal to the epicenter from NGF-NSCs treated, sham and control animals. Two weeks after SCI, the immunoactivity of nestin was spontaneously enhanced around the central canal, and its level decreased gradually at 2nd, 3rd, 4th, and 5th week after injury. In comparison, NGF-NSCs rats exhibited much higher and sustainable elevation of nestin expression without positive signals at the same anatomical site, which suggested NGF-NSCs transplantation could enhance the endogenous neurogenesis in the peri-lesion segments (two-way ANOVA, *p* < 0.05, Figures 5C–E).

### NGF-Modified NSCs Transplantation Simulated the Expression of Multiple Growth Factors Around Lesion Core

**FIGURE 6:**
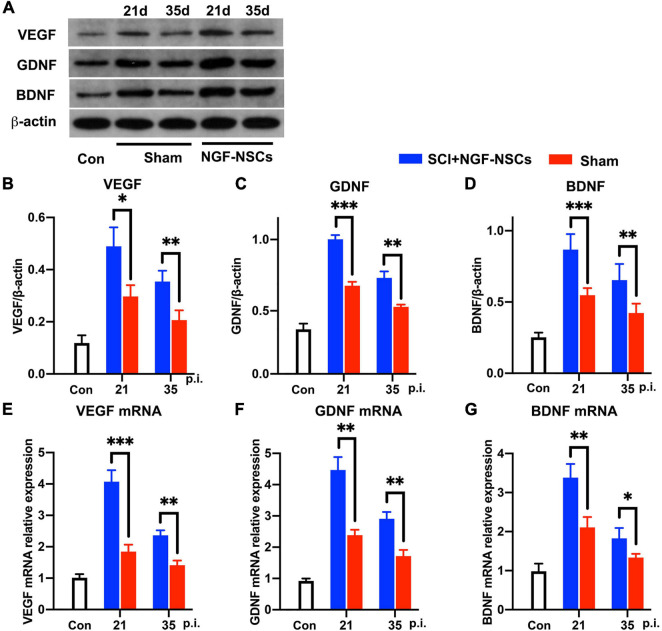
NGF-modified NSCs transplantation simulated the protein and mRNA levels of VEGF, GDNF, and BDNF around the lesion core. **(A–D)** At day 21 and day 35 post-injury, representative western blotting images demonstrated increased protein levels of VEGF **(B)**, GDNF **(C)**, and BDNF **(D)** in the NGF-NSCs group around the lesion core. **(E–G)** At the same time, the outcomes of RT-PCR exhibited elevated levels of VEGF mRNA **(E)**, GDNF mRNA **(F)**, and BDNF mRNA **(G)**, which validated our findings in western blotting. ^∗^*p* < 0.05, ^∗∗^*p* < 0.01, and ^∗∗∗^*p* < 0.001.

After SCI, growth factors [e.g., vascular endothelial-derived growth factor (VEGF), glial cell-derived neurotrophic factor (GDNF), and brain-derived neurotrophic factor (BDNF), etc] were reported to have pro-repair impact on the microenvironment for supporting spontaneous neurofunctional recovery (Richard and Sackey, 2021). Thus, we further evaluated the expression of multiple growth factors around the lesion core. Interestingly, both western blotting and RT-PCR analysis demonstrated that NGF-NSCs transplantation further enhanced the expression of VEGF (two-way ANOVA, *p* < 0.05, Figures 6A,B,E), GDNF (two-way ANOVA, *p* < 0.05, Figures 6A,C,F), and BDNF (two-way ANOVA, *p* < 0.05, Figures 6A,D,G) around lesion core at 2nd and 4th week after the treatment.

### NGF-Modified NSCs Transplantation Activated TrkA, Upregulated CREB and miR-132 in the Peri-Lesion Segments

**FIGURE 7:**
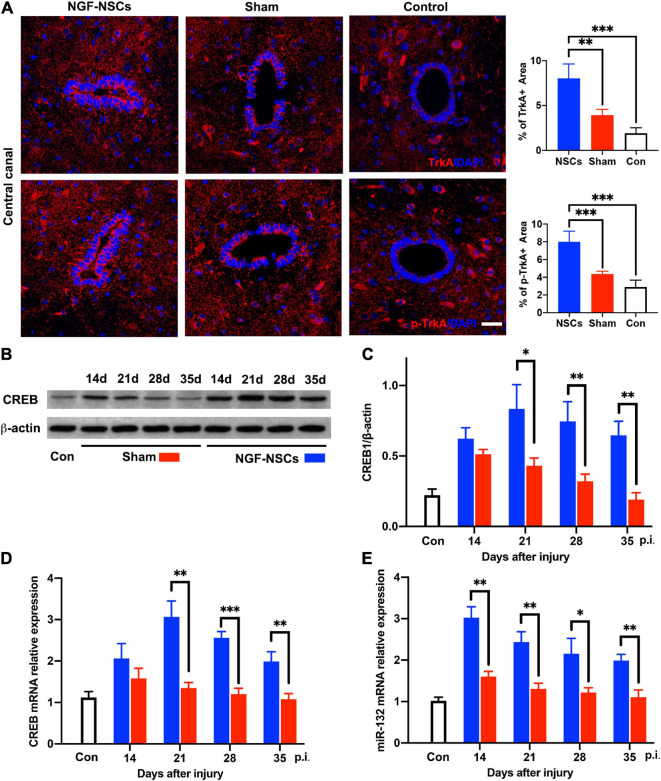
NGF-modified NSCs transplantation activated TrkA, upregulated CREB/miR132 in the peri-lesion segments. **(A)** In the NGF-NSCs group, the protein levels of TrkA and p-TrkA were significantly increased around central canal in the segment 3 mm caudal to the lesion site. **(B,C)** Compared to the control and sham rats, the protein level of CREB was significantly elevated in the NGF-NSCs group at day 21, 28, and 35 after injury. **(D)** Further RT-PCR examinations of CREB mRNA exhibited the same difference among the three groups along the time. **(E)** The levels of miR-132 in NGF-NSCs rats were significantly higher than that in the other two groups at day 21, 28, and 35 after injury. ^∗^*p* < 0.05, ^∗∗^*p* < 0.01, and ^∗∗∗^*p* < 0.001.

To elucidate the underlying mechanism of the neuroprotective effect of NGF-NSCs on SCI, spinal cord tissue samples at different time points from all three groups were selected for molecular analysis. Per previous knowledge, tropomyosin receptor kinase A (TrkA), cAMP-response element binding protein (CREB) and microRNA-132 (miR-132) were thought to be key mediators of the biological effect of NGF (Finkbeiner et al., 1997; Numakawa et al., 2011). In the peri-lesion segments (3 mm caudal to the lesion epicenter), the expression levels of TrkA were significantly increased at 4 weeks after NGF-NSCs transplants compared to the sham rats (2.0-fold, one-way ANOVA, *p* < 0.05, Figure 7A). The same increased expression pattern was observed for phospho-TrkA (p-TrkA) in the NGF-NSCs rats (1.8-fold, one-way ANOVA, *p* < 0.05, Figure 7A). Next, we evaluated the CREB expression via western blotting. Compared to the control rats, there was markedly up-regulation of CREB in both sham and NGF-NSCs groups (Figures 7B,C). Moreover, within the time of the experiments, the protein level of CREB was higher in the NGF-NSCs group (two-way ANOVA, *p* < 0.05, Figures 7B,C). Further RT-PCR examinations of CREB mRNA exhibited the same difference among the three groups along the time (two-way ANOVA, *p* < 0.05, Figure 7D). Then we examined the level of miR-132 at the same level along time. Similar to the CREB, we observed dramatic up-regulation of miR-132 in the SCI rats receiving NGF-NSCs (two-way ANOVA, *p* < 0.05, Figure 7E). Overall, the transplantation of NGF-NSCs could activated TrkA, upregulated CREB and miR-132 in the peri-lesion segments.

## Discussion

In this study, we genetically manipulated NSCs to overexpress NGF and evaluated its therapeutic value in contusive SCI. Our data indicate that (i) spinal cord-derived NSCs can be genetically modified to overexpress NGF while maintaining their stemness; (ii) in a rat SCI model, the transplantation of NGF-NSCs in the subacute stage can facilitate functional recovery; (iii) NGF-NSCs can survive and differentiate in the lesion core after the transplantation while sustainably expressing NGF; (iv) the graft of NGF-NSCs modulates the post-SCI microenvironment via reducing oligodendrocyte loss, attenuating astrocytosis and demyelination, preserving neurons, enhancing endogenous neurogenesis, and increasing the expression of various growth factors in peri-lesion segments; (v) the neuroprotective effect of NGF-NSCs may be mediated by the upregulation of CREB and miR-132.

As the first discovered member of neurotrophins, NGF plays a critical role in guiding axons, promoting axonal sprouting, and myelination of newborn axons after nerve injury (Yu et al., 2010; Tang et al., 2013). After neurotrauma in the spinal cord, endogenous NGF in the peri-lesion site is transiently upregulated within the first few weeks and contributes to the tissue repairment and local synaptic plasticity (Baldassarro et al., 2021). Therefore, an extra supplement of NGF may be a potential therapeutic strategy toward SCI. Initially, researchers tried to continuously infuse NGF directly into the lesion center by osmotic pumps, but the outcomes were inconsistent (Kobayashi et al., 1997; Bowes et al., 2000; Namiki et al., 2000). The problems of osmotic pumps were a potential failure of catheter insertion, limited time of use, and accidental secondary injury during the procedure, which introduced confounding factors into the experiments (Oladosu et al., 2016). Recently, Zhang et al. (2014) demonstrated in a rat SCI model that intravenous injection of NGF at a dose of 20 μg/kg/day could inhibit endoplasmic reticulum stress-induced apoptosis and improve the recovery after SCI. Via a nanocapsule-based delivery system, Xu et al. (2019) efficiently delivered the NGF into the CNS via intravenous administration, which subsequently slowed the lesion expansion and promoted tissue repair release in an acute SCI mice model. However, to sustainably elevate NGF at the lesion site, the animals had to receive a large dose of NGF and repeat injections, which might lead to systematic responses, inevitably subjected them to significant stress and messed up the behavioral assessment.

Recent advances indicate that genetically modified stem cells are ideal carriers to deliver specific molecules in the lesion site after spinal cord injury (Cui et al., 2013; Wei et al., 2018; Teng, 2019). Sasaki et al. (2009) genetically modified human mesenchymal stem cells to secret BDNF, and its transplantation restored the structural damage of the brain and spinal cord and contributed to improved functional outcomes in acute SCI. In comparison with other stem cells, NSCs exhibited their unique therapeutic values after neurotrauma, and the combination of neurotrophins and NSCs may have a synergistic effect on the recovery of SCI (Ruzicka et al., 2017; Han I. B. et al., 2019; Katoh et al., 2019). First of all, NSCs transplantation and neurotrophins administration have independent neuroprotective effects in the injured spinal cord (Zhao et al., 2019). Secondly, neurotrophins can influence the differentiation, enhance cell survival and proliferation of NSCs (Han and Kim, 2016; Langhnoja et al., 2021). Thirdly, the survival NSCs in the lesion site constitutively express neurotrophins, which ensure sustainable and high levels of neurotrophins in the microenvironment. For example, Khazaei et al. (2020) over-expressed GDNF in the human-induced pluripotent stem cell-derived NSCs. They observed that GDNF-expression biased the NSCs differentiation toward a neuronal fate via attenuating Notch signals *in vitro* and *in vivo*. And when transplanted into a rat model of cervical SCI, GDNF modified NSCs enhanced endogenous tissue repair, improved electrical integration of transplanted cells, and resulted in neurobehavioral recovery (Khazaei et al., 2020). Meanwhile, another research group tried to elevate the expression of BDNF in human NSCs, and their findings suggested that BDNF modified NSCs were able to attenuate neuroinflammatory response and glia activation following SCI (Sasaki et al., 2009). In our study, we successfully transplanted NGF-NSCs in the lesion area following SCI, and the treatment could maintain high levels of NGF in the injured spinal cord, which improves the post-injury recovery (Figures 1–3).

The inhibitive pathological microenvironment post-SCI is the key adverse factor affecting neurofunctional recovery after injury (Zhao et al., 2017; Cox et al., 2021). Following SCI, death of oligodendrocytes and profound demyelination results in the release of myelin-associated inhibitors, including myelin-associated glycoprotein, Nogo, and oligodendrocyte myelin glycoprotein, etc., which suppress axon regeneration (Boghdadi et al., 2018; Wang et al., 2020). Meanwhile, the activated microglias and infiltrating immune cells secrets high levels of proinflammatory cytokines while decreasing the expression of growth factors at the lesion center. In addition, the recruited and activated astrocytes within and around the lesion core deposit extracellular matrix proteins and form a dense fibrotic scar, which is another barrier to endogenous repair (Orr and Gensel, 2018). Thus, we further examined the related molecular markers in our study to determine whether NGF-NSCs can dampen the inhibitory microenvironment and facilitate the maturation of a pro-repair microenvironment. Notably, the graft of NGF-NSCs preserved the myelin sheath, reduced oligodendrocyte loss, attenuated astrocytosis, and maintained the physiological morphology of motor neurons in our study (Figures 4, 5). We also observed that multiple growth factors, such as GDNF, BDNF, and VEGF, were elevated after cell transplantation, suggesting that NGF-NSCs could modulate the microenvironment to exert a protective effect (Figure 6).

Additionally, NGF-NCSs also showed the ability to enhance endogenous neurogenesis around the central canal in our study (Figure 5). Recent evidence indicates that a population of ependymal cells lining the central canal of the spinal cord, namely the endogenous NSCs, restores its multipotency and starts dividing after SCI (Stenudd et al., 2015). However, in the pathologic status, endogenous NSCs generated more than half of the astrocytes and small amounts of oligodendrocytes, which mainly contributed to scar formation and manifested limited effect to functional recovery (Barnabé-Heider et al., 2010; Mao et al., 2016). Via integrating multimodal single-cell analysis, Llorens-Bobadilla et al. (2020) noticed that these endogenous NSCs were in a permissive chromatin state that could unfold latent gene expression and ectopic expression of the transcription factor OLIG2 promoted robust oligodendrogenesis, which suggested that modulation of resident NSCs might serve as an alternative treatment after SCI. Similarly, in our study, NGF-NSCs seemed to crosstalk with resident NSCs, and we thought this crosstalk was associated with continuously high levels of NGF and secondary up-regulation of various growth factors (e.g., GDNF, VEGF, and BDNF, etc.) via a paracrine method.

The transcription factor CREB is a critical downstream mediator of NGF-dependent gene expression in CNS, and its activation contributes to the neuroprotective effect of NGF (Riccio et al., 1999). It is known that NGF can bind to TrkA receptors on the cell surface, and this binding activates the Raf-MEK-ERK-RSK pathway, which subsequently activates CREB (Reichardt, 2006). Additionally, NGF-TrkA can also phosphorylate SH2Bβ and further activate ERK 1/2 and RSK, which participate in the activation of CREB (Maures et al., 2009). miR-132 expresses specifically in neural tissue, and CREB regulates its locus in response to neurotrophins (Wanet et al., 2012). Recently, miR-132 was reported to have a modulation effect on neuronal and immune function, which was designated as a “NeurimmiR” and suggested to act as crosstalk between both systems (Soreq and Wolf, 2011). Thus, we further evaluated the functional status of TrkA and the levels of CREB and miR-132 after cell transplantation. Notably, NGF- NSCs transplantation significantly activated TrkA, upregulated CREB and miR-132 in the peri-lesion segments, which suggests the protective effect of NGF-NSCs might be associated with their elevated expression.

Admittedly, there are several limitations in this study. First, the sensory tests, such as spinal sensory reflexes, von Frey filament tests, hot plane tests, etc., are not included in the behavior battery. Previously, the nociceptive sprouting induced by NGF and associated pain like behavior in the animal models limited the application of the neurotrophins in SCI treatment (Shu and Mendell, 1999). However, Tuszynski et al. (1996) observed that NGF delivery via fibroblasts elicited sprouting of local motor neurons, which was critical for the post-SCI recovery. Consistent with our findings, there are also emerging data demonstrating that NGF administration modulates the microenvironment, alleviates neuroinflammation, and facilitates the neural regeneration after SCI, which casts new light in the field of NGF (Cirillo et al., 2010; Xu et al., 2019). In addition, several researchers tried NSCs transplants to manage neuropathic pain treatment, and it significantly inhibited pain-like behavior after SCI (Yousefifard et al., 2016; Du et al., 2019). Thus, NGF and NSCs may have a synergic effect on post-SCI functional recovery without leading to chronic pain. It would be interesting for the future to explore the effect of NGF-NSCs on neuropathic pain after SCI. Second, as we did not include a group of LV-null NSCs in the animal study, there was no sufficient data showing that the NGF-NSCs could enhance the therapeutic efficacy of NSCs in SCI treatment. Third, in the molecular investigation, only three critical nodes (TrkA, CREB, and miR-132) in NGF-TrkA signaling were chosen for further analysis, and we did not use selective inhibitors and activators to elucidate the exact signaling pathway underlying the protective effect of NGF-NSCs. Fourth, the animals were sacrificed 4 weeks after cell transplantation in this study. Longer observation and evaluation of sensory and motor function should be performed to demonstrate the neuroprotective effect of NGF-NSCs in the chronic stage of SCI.

## Conclusion

In summary, our study demonstrates for the first time that NGF-NSCs can effectively reduce oligodendrocyte loss, attenuate astrocytosis and demyelination, preserve motor neurons, and activate endogenous neurogenesis in peri-lesion segments, and therefore improve the functional recovery after SCI. In addition, we found that the therapeutic effects of NGF-NSCs may be mediated by modulation of the microenvironment via activating TrkA, upregulation of CREB and miR-132 around the lesion core. Taken together, our findings may shed new light on the application of NGF and cell transplantation therapy in SCI.

## Data Availability Statement

The raw data supporting the conclusions of this article will be made available by the authors, without undue reservation.

## Ethics Statement

The animal study was reviewed and approved by Experimental Animal Care and Use Committee at Union Hospital, Tongji Medical College, Huazhong University of Science and Technology.

## Author Contributions

LW and DL: conceptualization, resources, and data curation. SG, JG, and YT: methodology and investigation. LW, SG, JG, and YT: formal analysis. LW, SG, and DL: writing—original draft preparation. LW, FZ, HZ, and DL: writing—review and editing. LW and SG: visualization. DL, FZ, and HZ: supervision. DL: project administration and funding acquisition. All authors have read and agreed to the published version of the manuscript.

## Conflict of Interest

The authors declare that the research was conducted in the absence of any commercial or financial relationships that could be construed as a potential conflict of interest.

## Publisher’s Note

All claims expressed in this article are solely those of the authors and do not necessarily represent those of their affiliated organizations, or those of the publisher, the editors and the reviewers. Any product that may be evaluated in this article, or claim that may be made by its manufacturer, is not guaranteed or endorsed by the publisher.

## Abbreviations

ANOVA: analysis of variance
BBB: Basso-Beattie-Bresnahan
CNS: central nervous system
GAPDH: glyceraldehyde 3-phosphate dehydrogenase
GDNF: glial cell-derived neurotrophic factor
miR-132: microRNA-132
NGF: nerve growth factor
NSCs: neural stem cells
PBS: phosphate buffered saline
SCI: spinal cord injury
SD: Sprague-Dawley
TrkA: tropomyosin receptor kinase A
VEGF: vascular endothelial-derived growth factor
BDNF: brain-derived neurotrophic factor.
