# Supplementary material for: Neural Stem Cells Overexpressing Nerve Growth Factor Improve Functional Recovery in Rats Following Spinal Cord Injury via Modulating Microenvironment and Enhancing Endogenous Neurogenesis
Source: Front Cell Neurosci. 2021 Dec 2;15:773375. doi: 10.3389/fncel.2021.773375 (PMC8675903; doi:10.3389/fncel.2021.773375)
Supplement: Supplementary file 1 [file Table_1.docx]

Supplementary Table S1: The primers used in qRT-PCR analysis.

| **mRNA/mi-RNA** | **Forward/Loop primer** | **Reverse/F primer** |
| --- | --- | --- |
| NGF | AGACCCGCAACATCACTG | CGTGGCTGTGGTCTTATCTC |
| VEGF | CACCAAAGCCAGCACATAGG | TTTAACTCAAGCTGCCTCGC |
| GDNF | AAATCGGGGGTGCGTCTTAACT | AACATGCCTGGCCTACCTTGTC |
| BDNF | TCTACGAGACCAAGTGTAATCCC | TATCCTTATGAACCGCCAGCCA |
| CREB | AACATACCAGATTCGCACAGC | ACGACATTCTCTTGCTGCTTC |
| GADPH | ACAGCAACAGGGTGGTGGAC | TTTGAGGGTGCAGCGAACTT |
| U6 | CGCTTCGGCAGCACATATAC | AAATATGGAACGCTTCACGA |
| miR-132 | GTCGTATCCAGTGCAGGGTCCGAGGTATTCGCACTGGATACGACCATGGTCG | TGCGCTAACAGTCTACAGCCAT |
